# Novel Slow-Release Defoamers for Concrete Using Porous Nanoparticles as Carriers

**DOI:** 10.3390/ma15227993

**Published:** 2022-11-12

**Authors:** Guangcheng Shan, Min Qiao, Jian Chen, Nanxiao Gao, Fei Shen, Qianping Ran

**Affiliations:** 1 State Key Laboratory of High Performance Civil Engineering Materials, Jiangsu Sobute New Materials Co., Ltd., Nanjing 211103, China; 2 Bote New Materials Taizhou Co., Ltd., Taizhou 225474, China; 3 Jiangsu Key Laboratory of Construction Materials, School of Material Science and Engineering, Southeast University, Nanjing 211189, China

**Keywords:** porous nanoparticles, defoamers, slow-release, concrete, bubbles

## Abstract

Excess large and unstable air bubbles can reduce the compressive strength of hardened concrete, and traditional defoamers always fail because of adsorption and encapsulation on cement with the progress of cement hydration in later stages. It is necessary to develop a novel defoamer that shows a sustained defoaming ability in fresh concrete. A novel slow-release defoamer for concrete using porous nanoparticles as carriers is reported for the first time. The porous nanoparticles/polyether defoamer composite (SiO_2_-Def) was prepared via sol-gel method. SiO_2_-Def is a spherical composite nanoparticle with a size range of 160–200 nm and a uniform pore size distribution. SiO_2_-Def shows a high load rate of about 16.4% and an excellent release under an alkali and salt environment. It has a weak initial defoaming ability but shows a sustained defoaming ability with time, so that it can avoid the failures of defoamers and eliminate harmful bubbles entrained during the processes of pumping and transportation. Moreover, SiO_2_-Def produced a higher compressive strength of the hardened cement mortars.

## 1. Introduction

Air entrainment effectively improves the durability and impermeability of concrete, meanwhile dramatically increasing the workability and eliminating the segregation and bleeding of fresh concrete [1,2,3,4]. However, the introduction of excess large and unstable air bubbles may reduce the compressive strength of hardened concrete, which is one of the most important factors in engineering applications [5]. In addition, with the development of the economy and the improvement of public aesthetics, requirements for the appearance of hardened concrete are becoming more strict. An excess of large air bubbles could induce a honeycomb-pockmarked surface of hardened concrete, which may cause a defective appearance and shorten the service life of a building [6,7]. Therefore, in order to solve the problem of honeycomb-pockmarked surfaces and to improve the mechanical performance and extend the service life of concrete, it is necessary to add defoamers to eliminate the excess air bubbles during the mixing, placing, and consolidation of concrete [8].

Defoamer is a class of surfactant with a specific hydrophilic–lipophilic balance (HLB) value, which could spread on the air–liquid interface of bubbles and lower surface tension. Defoamer can also disturb and destroy the mechanical equilibrium of the air–liquid interface of bubbles, thus inducing bubbles to break and inhibiting bubbles from forming. There are mainly four types of defoamers, which include mineral oils, silicones, polyethers, and polyether-modified silicones [9]. However, traditional defoamers show a strong defoaming ability in the initial mixing stage of fresh concrete, which is good for the mechanical strength of hardened concrete but not beneficial to the workability of fresh concrete. On the other hand, the mechanical strength of hardened concrete without the addition of defoamers can hardly meet the necessary requirements. In addition, traditional defoamers always lose their effectiveness with the progress of cement hydration in the later stage, which is a result of the adsorption and encapsulation on cement [10]. Therefore, it is of great significance to develop a novel defoamer that shows weak defoaming ability only in the initial mixing stage and a sustained reduction in the air contents of concrete. A defoamer with this property can also improve the compressive strength of concrete and extend its service life, e.g., as in ultrahigh performance concrete (UHPC), bridge concrete, and fair-faced concrete.

Slow-release technology is an important method to improve the time-dependent behavior of materials. Here, an active substance is usually in a carrier, and the active substance is slowly released with time. There are many carriers to be chosen for different purposes, such as super-absorbent polymers (SAP) [11,12,13], microcapsules [14,15,16], and porous nanomaterials [17,18,19]. Among these, porous silica nanoparticles have been widely used in many fields due to their high specific surface area and large pore volume [20]. Slow-release technology is currently widely applied in the fields of medicine [21,22], oil [20,23], food [24,25,26], and so on. In the field of concrete, it has been applied in the performance control of cement-based materials, such as internal curing, crack self-repair [11,12,13,14,15,16], expansion control, and rust inhibition [27,28]. However, studies have rarely reported on the use of slow-release technology for the time-dependent defoaming of concrete.

**Figure 1:**
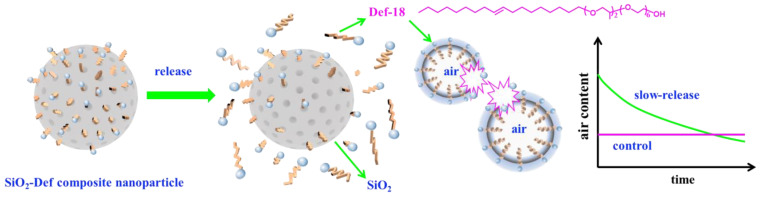
Illustration of the slow-release process of SiO_2_-Def.

In this work, a novel slow-release defoamer for concrete using porous nanoparticles as the carrier is reported for the first time. The preparation of the slow-release defoamers (named SiO_2_-Def in this paper) and the overall slow-releasing strategy is schematically illustrated in Figure 1. Defoamer molecules are released from SiO_2_-Def to act on the surface of air bubbles, and they exhibit a slow-release effect. Compared to the control sample, the slow-release defoamer exhibits a sustained defoaming effect for the air bubbles of fresh concrete. SiO_2_-Def was prepared via the sol-gel method [29,30], which used a polyether defoamer (Def-18) as the templating agent and tetraethyl orthosilicate (TEOS) as the silicon source. The obtained SiO_2_-Def has a well-distributed diameter and uniform pore size distribution. The defoamer load rate of SiO_2_-Def is about 16.4%, and the cumulative release amount can be enhanced by alkali and salt [31], reaching a maximum of 12%. SiO_2_-Def has poor defoaming ability in the initial mixing stage of fresh cement mortars but a sustained defoaming ability with the passage of time. In addition, SiO_2_-Def contributed mechanical strength to the hardened cement mortars.

## 2. Materials and Methods

### 2.1. Materials

Tetraethyl orthosilicate (TEOS), ammonia (NH_3_·H_2_O), and ethanol (C_2_H_5_OH) were purchased from Sigma-Aldrich (St. Louis, MO, USA). Sodium hydroxide (NaOH), sodium chloride (NaCl), and calcium chloride dehydrate (CaCl_2_·2H_2_O) were obtained from Sinopharm Chemical Reagent Co., Ltd., (Shanghai, China). The defoamer used in this study was a polyether with a C18 hydrophobic chain (Def-18). A high-performance polycarboxylate superplasticizer (PCA) was synthesized by the polymerization of acrylic acid and alkenyl polyoxyethylene ether using free radical polymerization, and it had an average molecular weight of 40,000. The dosage of PCA was adjusted according to the test requirements. Def-18 and PCA were obtained from Jiangsu Sobute New Materials Co., Ltd. (Nanjing, China). PII52.5 Portland cement was obtained from Jiangnan-Xiaoyetian Cement Co., Ltd., (Nanjing China), and its chemical composition and mineral composition are summarized in Table 1. Chinese ISO standard sand with a nominal grain size of 0.08–2 mm was used as fine aggregate.

### 2.2. Preparation of SiO_2_-Def

**Figure 2:**
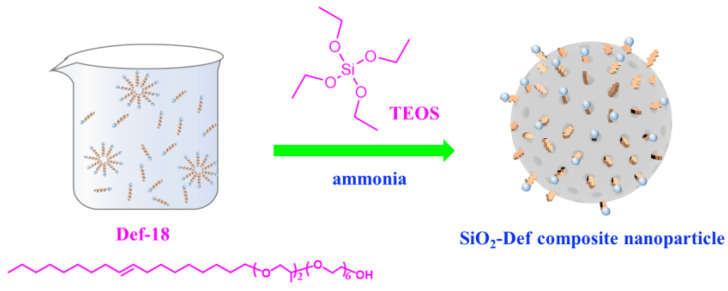
The preparation procedure of SiO_2_-Def nanoparticles.

SiO_2_-Def was prepared via the sol-gel method using Def-18 as the templating agent. TEOS as the silicon source was hydrolyzed into silica nanoparticles under alkaline conditions. Silica grew along with the templating agent into the porous nanoparticles/polyether defoamer composite. The preparation procedure of SiO_2_-Def is shown in Figure 2. An amount of 85 g defoamer and 5 g NH_3_·H_2_O (28 wt%) were completely mixed in a three-necked flask at 30 °C. Subsequently, 10 g TEOS (dissolved in 10 g defoamer) was added slowly to the mixture within 4 h for the sol-gel reaction. Then, the mixtures were stirred continuously at room temperature in a flask for another 5 h. The product was then centrifuged and washed with 50 g C_2_H_5_OH to remove the unadsorbed defoamer. After drying under vacuum, SiO_2_-Def was obtained as a white power. In addition, pure silica nanoparticles without defoamers (SiO_2_) were prepared under the same conditions.

### 2.3. Characterization of SiO_2_-Def

The morphological structure of SiO_2_-Def nanoparticles was characterized by scanning electron microscopy (SEM, Nova Nanosem450, FEI Company, Hillsboro, MO, USA) and transmission electron microscopy (TEM, TencnaiG2F20, FEI Company, Hillsboro, MO, USA). The specimens were gold-coated prior to examination. The hydrodynamic size distribution and particle dispersion index (PDI) of the SiO_2_-Def nanoparticles, which were dispersed in ethanol solutions, were tested by dynamic light scattering (DLS, CGS-3, ALV, Langen, Germany). The chemical structure of the nanoparticles was tested by Fourier transform infrared spectra (FTIR, Nicolet iS20, Thermo Fisher Scientific, Waltham, MA, USA), with a spectral range of 500–4000 cm^−1^ and resolution of 0.02 cm^−1^. Thermogravimetric analysis (TGA, TGA 550, TA, New Castle, DE, USA) was used to quantify the organic content present, with a heating rate of 10 °C min^−1^ from room temperature to 900 °C. The instrument has an isothermal accuracy of ±1 °C. The samples were dried adequately at 105 °C, and about 10 mg of the powder was placed on the platinum pan and loaded into the TGA. The nitrogen adsorption–desorption curve, specific surface area, pore volume, and pore size distributions of SiO_2_-Def were measured by an automated surface area and pore size analyzer (BET, TriStarII3020, Micromeritics, Atlanta, GA, USA), with samples degassed at 120 °C for 2 h prior to analysis.

### 2.4. Total Organic Carbon Measurement

The slow-release results of the nanoparticles in solutions were obtained by total organic carbon analyzer (TOC, Multi N/C3100, Analytik Jena, Jena, Germany). An amount of 0.5 g of SiO_2_-Def was dispersed in 50 g of solution with a different alkalinity (pH 7, 10, or 13) or various concentrations of salt (Na^+^: 0.01, 0.03, 0.06 M; Ca^2+^: 0.0028, 0.00625, 0.0125 M) and then stirred with high speed. The suspensions (3 g) were drawn out from the release system at different stirring times (0, 5, 15, 30, 60, 120, and 150 min, respectively) and then filtered. Two g of filtrate was collected, and then 1 g of HCl solution (1 M) was added to remove the inorganic carbon materials, diluted 6 times with deionized water, and then the mixtures were analyzed for the organic carbon contents released in the solutions by a TOC analyzer. A standard curve of organic carbon contents with different concentrations of defoamers was also developed. Then, the concentration of slow-release defoamer was read from the standard curves to calculate the amount of defoamer released per unit of SiO_2_-Def.

### 2.5. Fresh Cement Mortars Measurement

The fresh-cement mortar mixing procedure was in accordance with the Chinese National Standard GB/T 17671-1999. The corresponding mortar mixtures consist of 675 g cement, 1350 g Chinese ISO standard sand (the sand/cement ratio was set at 2:1), a required amount of PCA superplasticizer solution (10%), a predetermined amount of water (the water/cement mass ratio (*w*/*c*) was set at 0.4), and 0.1 g SiO_2_-Def. The quartz content of Chinese ISO standard sand is ≥96%, the fineness modulus is 3.0, the mud content is 0.05%, and it is composed of 1.0–2.0 mm, 0.5–1.0 mm, and 0.08–0.5 mm sand mixed by the mass ratio 1:1:1. After stirring, the fresh cement mortars were poured in a SANYO direct reading air content tester (SANYO, Osaka, Japan) to obtain the air content of the mortars, and another portion was taken out for the bubble distribution of the tested fresh mortar. The remaining fresh cement mortars were stirred and tested after 0.5, 1, 2, and 3 h, respectively.

The bubble distribution of the fresh mortar was tested using the fresh concrete pore structure analyzer (AVA3000, Germann Instruments, Copenhagen, Denmark). Detailed test methods and principles refer to the procedure in the literature [32]. Meanwhile, the air content and the bubble distribution of the fresh mortars containing Def-18 (the initial adding amount was 0.012 g) and the control sample without SiO_2_-Def were also tested.

### 2.6. Hardened Cement Mortar Measurement

The fresh cement mortars were incubated for 14 days to obtain hardened cement mortar cubic specimens (100 mm × 100 mm × 100 mm). These specimens were cut into thick slices whose thickness was 1.5 ± 0.2 cm, and then they were ground, buffed, and washed by a grinding and polishing machine (MP-260E, Laizhou Metallographic Testing Equipment Co., Ltd., Yantai, China), successively. Next, the slices were used to measure the air-void parameters of the hardened cement mortars by a hardened concrete pore structure analyzer (NELD-BS630, NELD, Beijing, China). The air content, air-void spacing factor, and air-void images of all the slices were recorded, and the results were averaged for triplicate measurements.

The fresh cement mortars were also incubated at 3, 7, and 28 days to obtain hardened cement mortar specimens (40 mm × 40 mm × 160 mm) and placed in the mortar curing room (temperature: 20 ± 1 °C, relative humidity: 90 ± 0.5%). The compressive strength of the hardened cement mortar specimens was measured by the mortar strength-testing machine (AEC-201, AEC, Shanghai, China).

## 3. Results and Discussion

### 3.1. Characterization of SiO_2_-Def

**Figure 3:**
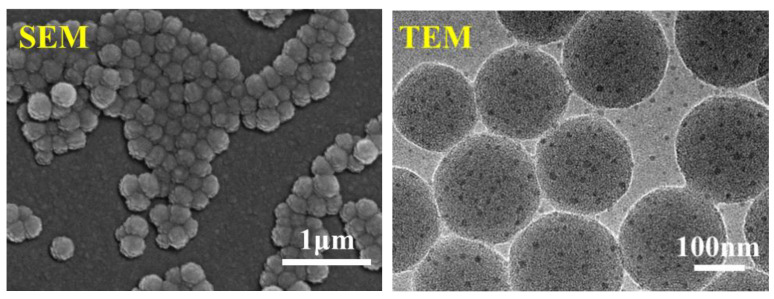
SEM and TEM images of SiO_2_-Def.

The morphology and structure of the SiO_2_-Def nanoparticles were initially characterized. SEM and TEM images of SiO_2_-Def are shown in Figure 3. The morphology of SiO_2_-Def shows that SiO_2_-Def is spherical, monodisperse, and has a uniform particle size distribution with an average diameter of about 160-200 nm. The results indicated that the spherical composite nanoparticle SiO_2_-Def was successfully prepared.

**Figure 4:**
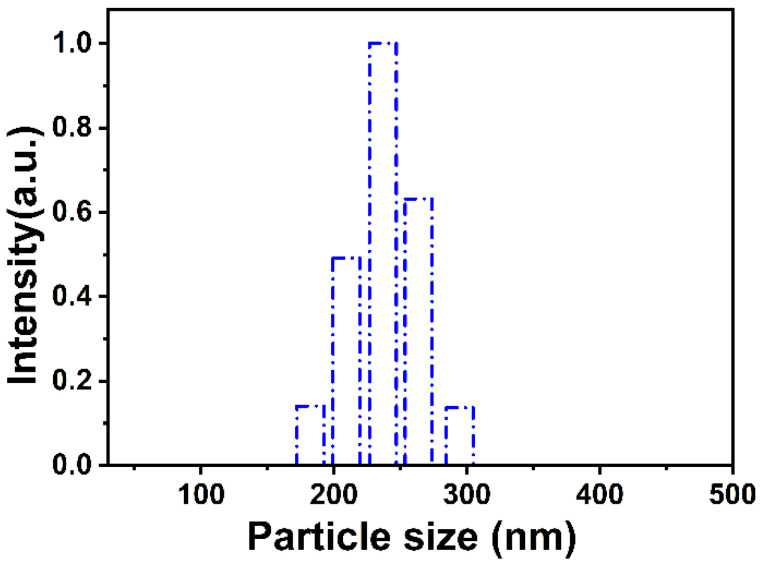
Size distributions (DLS) of SiO_2_-Def nanoparticles.

Figure 4 shows the hydrodynamic size distribution and dispersity of the SiO_2_-Def nanoparticles. The particle size distribution of SiO_2_-Def tested by DLS ranged from around 180 to 290 nm, and the nanoparticles had excellent dispersibility, indicating that the prepared SiO_2_-Def has a high dispersity and uniform particle size distribution. It is worth pointing out that the diameter of the nanoparticles tested by DLS was slightly larger than that which is shown in the SEM and TME images, which is caused by the occurrence of aggregation between particles or partial doublet formation through fusion in suspensions [33,34].

**Figure 5:**
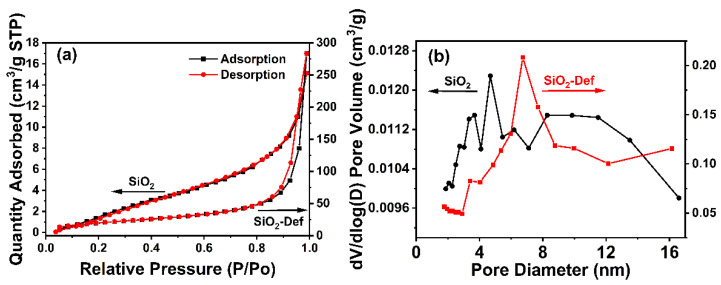
Nitrogen adsorption isotherms (**a**) and pore size distribution curves (**b**) of SiO_2_ and SiO_2_-Def (STP means standard temperature and pressure, the arrows point to the vertical coordinates of each side).

The nitrogen adsorption isotherms and pore size distribution curve of SiO_2_ and SiO_2_-Def, calculated by the Barrett–Joyner–Halenda (BJH) method [35,36], are shown in Figure 5, indicating that SiO_2_ and SiO_2_-Def nanoparticles have comparatively uniform porous structure, and the adsorption isotherms all exhibit type III. Table 2 reports the porous textural properties of SiO_2_ and SiO_2_-Def, respectively. As we know, the specific surface area and pore volume of nanoparticles play important roles in slow-release technology; a larger specific surface area and pore volume could improve the activity of particles and provide more active sites to adsorb and store more defoamer molecules [37]. The specific surface area, pore volume, and pore diameter of SiO_2_-Def were larger than that of SiO_2_, which indicated that the prepared SiO_2_-Def had sufficient capacity to make wide contact with defoamer molecules and increase the loading amount.

**Figure 6:**
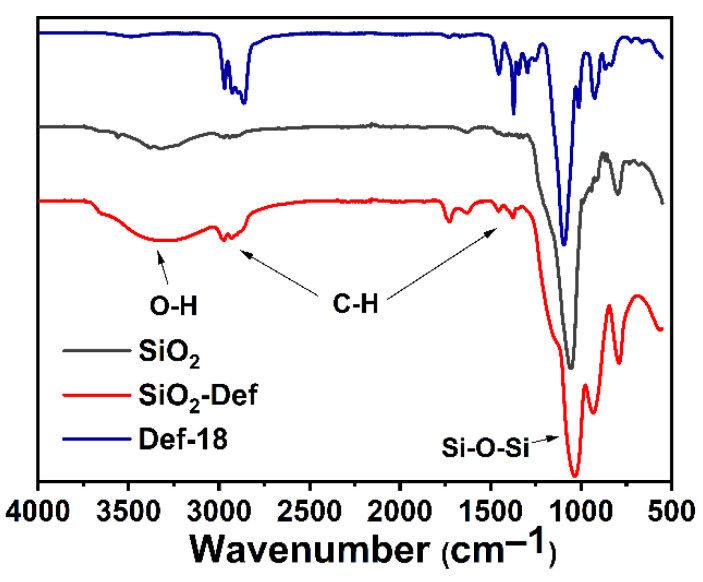
FT-IR spectra of SiO_2_, Def-18, and SiO_2_-Def.

The FT-IR spectra of SiO_2_, SiO_2_-Def, and Def-18 are given in Figure 6. In all the spectra, the characteristic peaks of SiO_2_ were related to the stretching vibration at 1070 cm^−1^ and the bending vibration at 800 cm^−1^ of the Si-O-Si bond, respectively. The strong absorption bands at 2926, 2857, and 1411 cm^−1^ represent the stretching and bending vibration of the C-H bond in the -CH2-CH2- group belonging to Def-18. As shown in the spectra of SiO_2_-Def, upon treatment with SiO_2_ and Def-18, the bending vibrations at 1070, 800, 2926, 2857, and 1411 cm^−1^ were all observed. In addition, the wide peaks at 3400–3500 cm^−1^ represent the stretching vibration of the -O-H based on the adsorbed and crystal water of the air or SiO_2_-Def nanoparticles, which can be disregarded. The above results clearly confirm the successful preparation of SiO_2_-Def.

**Figure 7:**
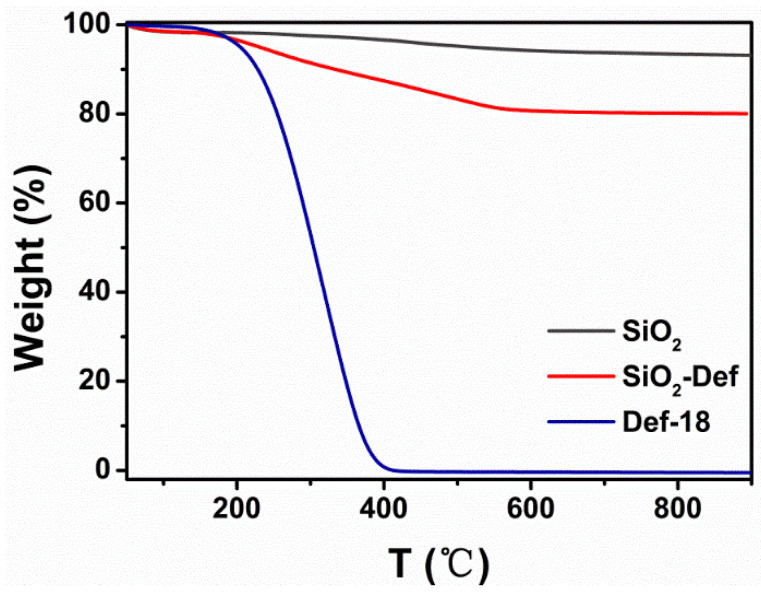
TGA curves of SiO_2_, Def-18, and SiO_2_-Def.

To further roughly confirm the loading amount of defoamers, the TGA of SiO_2_, SiO_2_-Def, and Def-18 was performed, which is shown in Figure 7. The weight of Def-18 started to decrease from 200 °C, and the weight loss of Def-18 reached about 100% until the temperature reached 400 °C because of the thermal decomposition. The weight loss of SiO_2_ was about 6.8% from 200 to 600 °C due to the dehydroxylation of Si-OH on the surface of silica, and the weight loss of SiO_2_-Def was about 20% due to a combination of the decomposition of organic molecules and the dehydroxylation of Si-OH. The loading rate (*W*t) of the defoamers in the SiO_2_ nanoparticles was about 16.4%, which was calculated by the following Equation (1):

(1) $$W_{t}=\frac{M_{3}-M_{1}}{M_{2}-M_{3}}$$

where *M*_1_, *M*_2,_ and *M*_3_ are the weight loss of SiO_2_, Def-18, and SiO_2_-Def, respectively. The *M*_1_, *M*_2,_ and *M*_3_ values are 6.8%, 100%, and 20%, respectively.

### 3.2. The Slow Release of SiO_2_-Def Nanoparticles in Solution

In order to study the slow-release behavior of SiO_2_-Def, the released amount of the defoamers from SiO_2_-Def in different environments at different times was measured by TOC.

**Figure 8:**
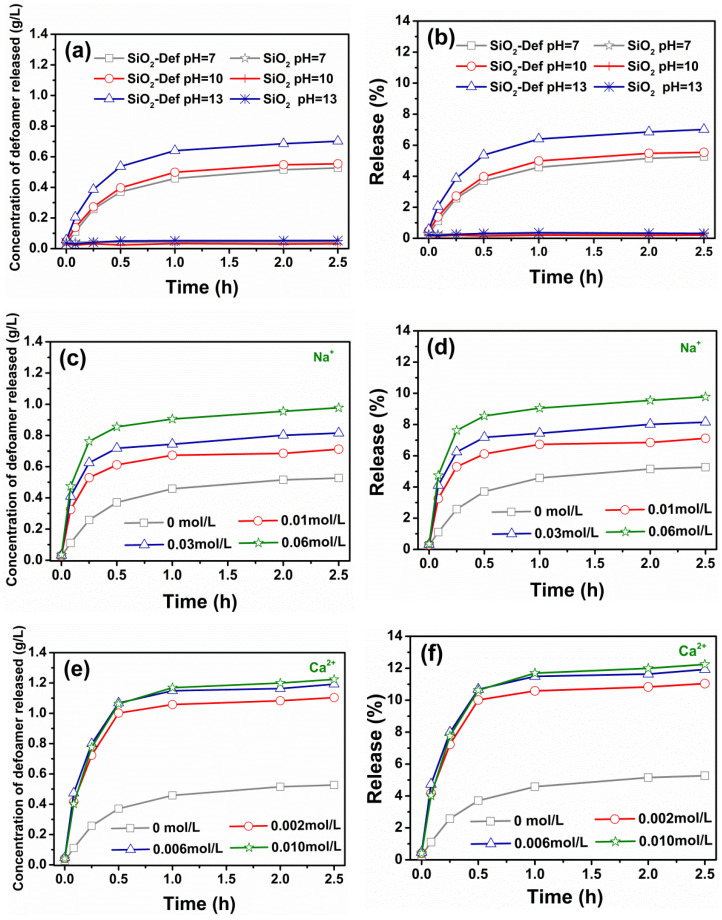
Release profile of SiO_2_-Def under different conditions: (**a**,**b**) different pH; (**c**,**d**) different concentrations of Na^+^; (**e**,**f**) different concentrations of Ca^2+^.

The release profiles of SiO_2_-Def at different pH values within 2.5 h were initially studied. Figure 8a shows the concentration of defoamer released as a function of time, which was read from the standard curve. Figure 8b shows the cumulative released amount of defoamer per unit of SiO_2_-Def, calculated from Figure 8a, which was the released rate. As can be seen, the released profiles of SiO_2_-Def all increased rapidly with increasing time over 1 h, but the growth rate slowed down in the next 1.5 h. In other words, the speed of the defoamers released showed the trend of increasing and then decreasing. Significantly, the released amount of defoamers from SiO_2_-Def increased with the increase in the pH values. Silica is an acidic oxide, which reacts easily with alkalis and then decomposes, thus improving and accelerating the release of the loaded defoamer molecules [31,38]. The total cumulative released amount at pH 7 was 5.3%, and at pH 10 and pH 13, it was 5.5% and 7.0%, respectively, indicating that increased alkalinity enhanced defoamer release. Consequently, SiO_2_-Def has a certain release property based on pH response, which can be useful as a novel slow-release defoamer in concrete.

In addition to a high-alkaline environment, concrete also has a high salt concentration, and the release of SiO_2_-Def in a salt environment also needs to be considered. Figure 8c–f shows the release profiles of SiO_2_-Def as a function of time under different concentrations of Na^+^ and Ca^2+^, respectively. The released amount of defoamers all increased at different salt concentration solutions compared with that in pure water. The total cumulative released amount gradually increased with the concentration of Na^+^ in the solutions, and the highest released amount was 9.8%. Further, Ca^2+^ was also beneficial to the release of defoamers, although the release did not increase significantly with the increase in the concentrations set. Its maximum released amount reached 12 wt% of SiO_2_-Def. All the above results indicate that a high-salt and -alkaline environment promoted the release of defoamers from SiO_2_-Def.

### 3.3. Applications of SiO_2_-Def for Fresh Cement Mortars

**Figure 9:**
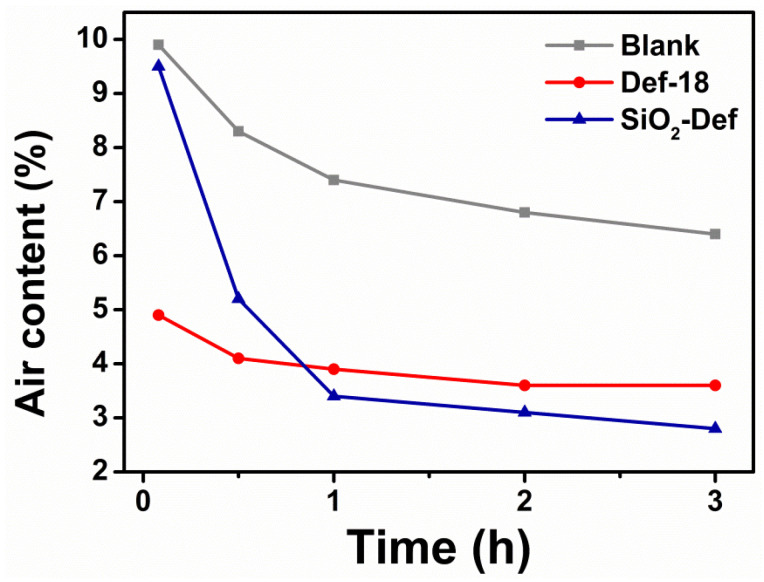
Air content (%) of blank, Def-18 and SiO_2_-Def in fresh cement mortars.

The slow release of SiO_2_-Def in cement mortars was further investigated. As shown in Figure 9, the initial air content of the reference sample Def-18 was significantly lower than that of the blank sample, and it decreased slightly with time. By contrast, the initial air content of SiO_2_-Def was almost the same as that of the blank and markedly decreased with increasing time. After 3 h of incubating time, the air content of SiO_2_-Def was lower than the blank sample. SiO_2_-Def had only a slight defoaming ability in the initial stage, but with the progress in cement hydration, the slow release of defoamer molecules from SiO_2_-Def was enhanced due to the highly salty and alkali environment [38]. These results indicate that SiO_2_-Def has an ability to decrease the air contents by the continuous release of Def-18 in cement mortars.

**Figure 10:**
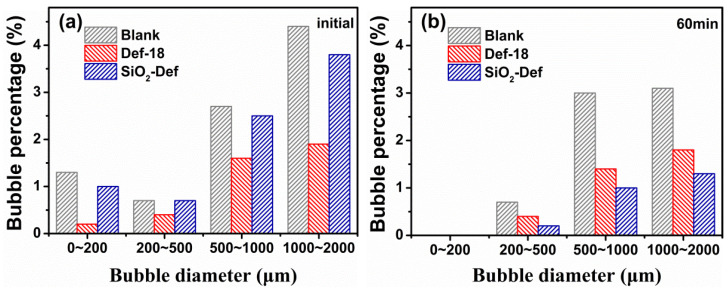
Effect of Def-18 and SiO_2_-Def on the bubble size distribution of the fresh mortar ((**a**) initial, (**b**) 60 min).

The bubble size distributions of cement mortars containing Def-18 and SiO_2_-Def were also measured. Figure 10a shows that at the beginning, the blank sample entrained a larger amount of bubbles with the size distributed between 0~2000 μm. By contrast, Def-18 obviously had less bubbles compared with the blank, and SiO_2_-Def had slightly less bubbles than the blank. Furthermore, Figure 10b shows that the bubble amount of SiO_2_-Def was significantly less than that of the blank and Def-18 after 60 min. This also suggests that SiO_2_-Def can slowly release the defoamer, and it shows a weak defoaming ability in the initial stage and a sustained defoaming ability with time in fresh concrete. Compared with traditional defoamers [39,40], SiO_2_-Def has a slow-release property, which improves the efficiency of defoamers. A sustained defoaming can avoid the failure of defoamers caused by the adsorption and encapsulation on the cement in the later stage [10].

### 3.4. Applications of SiO_2_-Def in Hardened Cement Mortars

**Figure 11:**
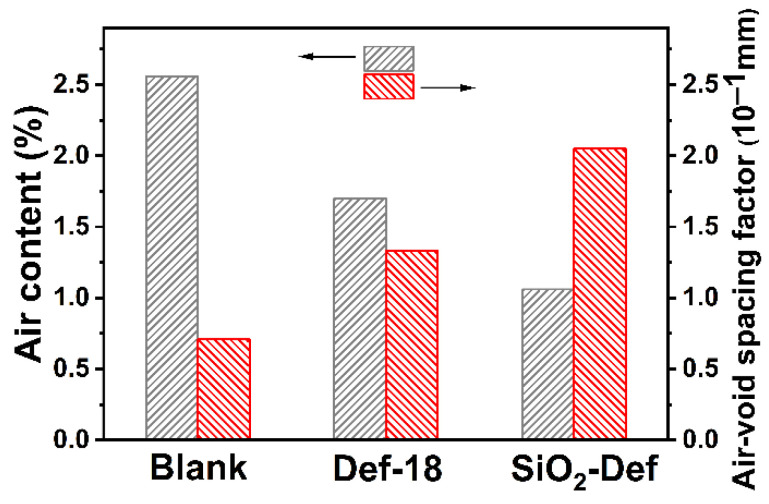
Air content and air-void spacing factor of the hardened cement mortar specimens containing Def-18 and SiO_2_-Def.

The air-void parameters (air content and air-void spacing factors) of SiO_2_-Def in hardened cement mortars, as shown in Figure 11, were also tested. The air content of hardened cement mortar specimens containing SiO_2_-Def after incubating for 14 days was significantly lower than that of the blank and Def-18 samples, whereas in contrast, the trend of the air-void spacing factors was higher. The results indicated that air voids in the hardened cement mortars induced by SiO_2_-Def are relatively fewer compared with that of the blank and Def-18 samples, which is highly consistent with the results of the air content and bubble size distribution tested in fresh cement mortars.

**Figure 12:**
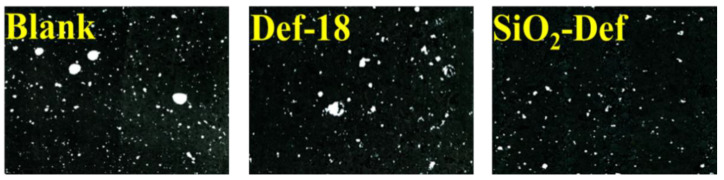
Air-void images of the hardened cement mortar specimens of blank, Def-18, and SiO_2_-Def.

The photograph of each hardened cement mortar specimen was taken to directly observe the air-void structure. Figure 12 shows the surfaces of the hardened cement mortar specimens. Among them, the blank and Def-18 samples had a number of non-homogeneous air voids. By contrast, SiO_2_-Def had a smooth surface of relatively few air voids. These results fully suggest that SiO_2_-Def has beneficial effects on the appearance of hardened concrete.

**Figure 13:**
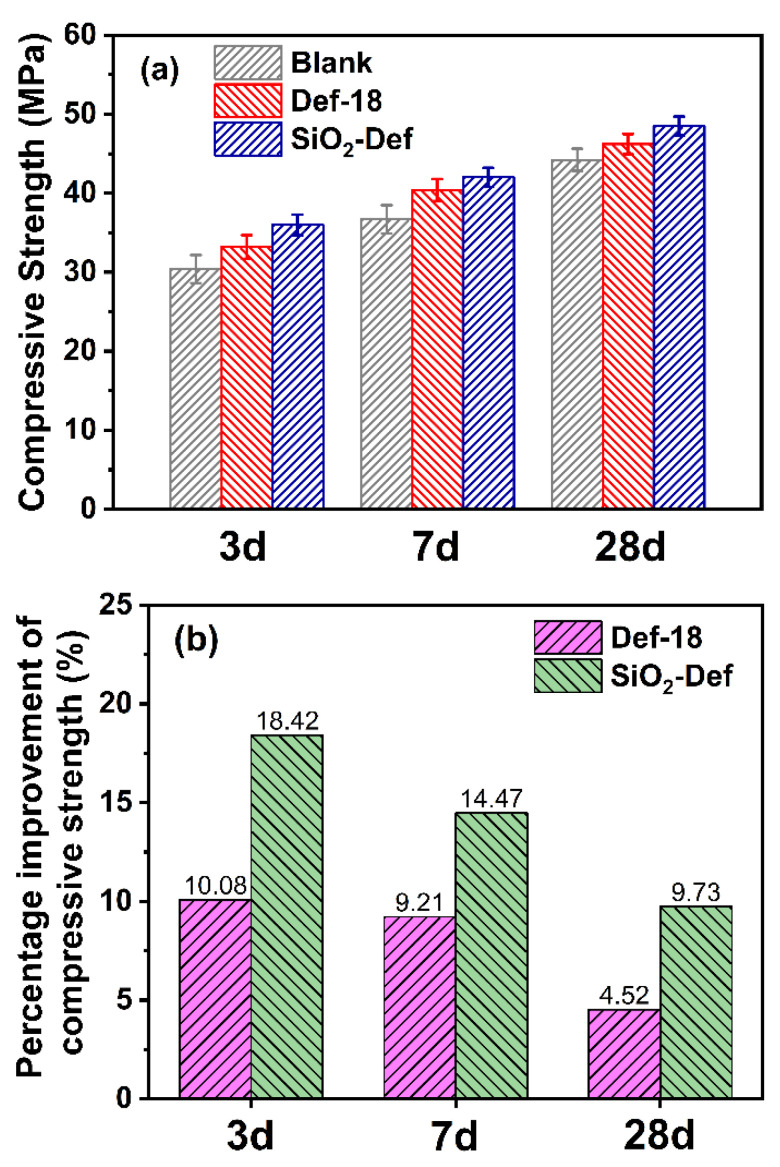
Compressive strengths (**a**) and percentage improvement of compressive strengths (**b**) of the hardened cement mortar specimens of Def-18 and SiO_2_-Def.

The mechanical strengths of hardened cement mortars containing samples after incubating for 3, 7, and 28 d are shown in Figure 13a. It was observed that the compressive strength of the hardened mortars containing SiO_2_-Def and Def-18 specimens was obviously higher than those of the blank specimen. Figure 13b shows that SiO_2_-Def has a higher percentage improvement of compressive strength than the Def-18 specimen at 3, 7, and 28 d. In other words, SiO_2_-Def has an obvious positive effect on the short-term and long-term compressive strength of the hardened mortars. Therefore, SiO_2_-Def was selected to avoid the failure of defoamers caused by adsorption and encapsulation on cement and to eliminate such harmful bubbles entrained during the processes of pumping and transportation. The reduced air content improved the compressive strength of mortars in the later stage, which shows excellent performance in both fresh and hardened cement mortars. In addition, silica has pozzolanic activity, which can react with calcium hydroxide in cement to generate calcium silicate hydrate and calcium aluminate hydrate, contributing to the development of cement hydration and improving the compressive strength [41,42].

## 4. Conclusions

In summary, a porous nanoparticles/polyether defoamer composite was evaluated as a novel slow-release defoamer for concrete for the first time. SiO_2_-Def was successfully prepared by the sol-gel method, and the morphological structure, size distribution, and pore size distribution of SiO_2_-Def were characterized. The loading rate of defoamers and cumulative released amount of SiO_2_-Def were measured. The utility of SiO_2_-Def in novel slow-release defoamers in both fresh and hardened cement mortars was also studied. The following important conclusions were obtained from our study. First, the obtained SiO_2_-Def is a spherical nanoparticle with a size range of 160–200 nm. It has uniform particle size distribution and homogeneous pore size distribution, a high surface area, and large pore volume. Second, the defoamer load rate of SiO_2_-Def is about 16.4%, and the cumulative released amount is enhanced by alkali and salt, reaching a maximum of 12%. Third, SiO_2_-Def has poor initial defoaming ability, which is a benefit to the initial workability of concrete, but a sustained defoaming ability with time, so that it can avoid the failure of defoamers caused by adsorption and encapsulation on cement and eliminate harmful bubbles in the later stage. Fourth, the hardened cement mortar specimen containing SiO_2_-Def has less air voids and also exhibits a higher compressive strength. Finally, our study shows the great potential of SiO_2_-Def for concrete applications, which may also help provide a reference for the research and development of slow-release materials and techniques for better performance in concrete, such as UHPC, bridge concrete, and fair-faced concrete. This technology could improve the mechanical properties of concrete, especially the compressive strength, which helps to extend the service life of concrete.

## Figures and Tables

**Table 1:** Chemical analysis and mineral composition of cement.

**Chemical Composition**	**SiO_2_**	**Al_2_O_3_**	**CaO**	**MgO**	**Fe_2_O_3_**	**SO_3_**	**K_2_O**	**Na_2_O**
(% by mass)	19.61	3.13	65.12	0.68	3.13	2.32	0.68	0.16
**Mineral Composition**	**C_3_S**	**C_2_S**	**C_3_A**	**C_4_AF**	**CaSO_4_·xH_2_O**	**CaCO_3_**		
(% by mass)	62.0	12.9	6.4	8.8	5.5	4.3		

**Table 2:** Specific surface areas, pore volumes, and pore diameters of SiO_2_ and SiO_2_-Def.

Sample	Specific Surface Area (m^2^ g^−1^)	Pore Volume (cm^3^ g^−1^)	Pore Diameter (Å)
SiO_2_	10.6307	0.01003	46.279
SiO_2_-Def	73.4977	0.10942	63.512

## Data Availability

The data are contained within the article. Additional data are available upon request from the corresponding author.
